# Harnessing Transcription Factors as Potential Tools to Enhance Grain Size Under Stressful Abiotic Conditions in Cereal Crops

**DOI:** 10.3389/fpls.2020.01273

**Published:** 2020-08-18

**Authors:** Calum Watt, Gaofeng Zhou, Chengdao Li

**Affiliations:** Western Crop Genetics Alliance, Murdoch University, Perth, WA, Australia

**Keywords:** transcription factor, abiotic stress, grain size, rice, wheat

## Abstract

Predicted climate change is widely cited to significantly reduce yields of the major cereal crop species in a period where demand is rapidly rising due to a growing global population. This requires exhaustive research to develop genetic resources in order to address the expected production deficiencies which will largely be driven by abiotic stress. Modification of multiple genes is an approach that can address the predicted challenges; however, it is time-consuming and costly to modify multiple genes simultaneously. Transcription factors represent a group of proteins regulating multiple genes simultaneously and are therefore promising targets to concurrently improve multiple traits concurrently, such as abiotic stress tolerance and grain size (a contributor to yield). Many studies have identified the complex role that transcription factors of multiple families have contributed toward abiotic stress tolerance or grain size, although research addressing both simultaneously is in its infancy despite its potential significance for cereal crop improvement. Here we discuss the potential role that transcription factors may contribute toward improving cereal crop productivity under adverse environmental conditions and offer research objectives that need to be addressed before the modification of transcription factors becomes routinely used to positively manipulate multiple target traits.

## Introduction 

Rice, maize, and wheat collectively represent the primary food source for over half of the world’s population and are thus crucially important for global food security. However, with increased demand from a growing global population and an increasing fluctuation in crop productivity driven by climatic variability, food security is becoming increasingly vulnerable. Modest increases of 1°C in average growing season temperature for instance has been predicted to reduce global wheat yields anywhere between 4 and 8%, less for rice but greater yield losses are expected for maize, barley, and sorghum (Challinor et al., 2014; Liu B. et al., 2016; Zhao C. et al., 2017; Abhinandan et al., 2018). Already average temperature increases between 1981 and 2002 of ~0.4°C have resulted in estimated combined annual yield losses of 42 Mt for wheat, barley, and maize (Lobell and Field, 2007). Alternatively, the frequency of drought is projected to intensify, and 20% of global agricultural land is affected by salinity stress that is expected to double by 2050 both of which are considered to be the two primary threats to future agriculture (Lesk et al., 2016; Majeed et al., 2019; Nutan et al., 2019b). Our ability to ensure food security in the face of these threats therefore lies in our ability to improve cereal yields which in turn are reflections of two major and genetically manipulable morphological components: (1) number of grains/m^2^ and, (2) individual grain weight (Xing and Zhang, 2010; Kennedy et al., 2016; Li et al., 2018; Ji et al., 2019). Individual grain weight is a reflection of grain size which is the culmination of complex biological processes and pathways controlled by polygenes acting pre- and post-anthesis to determine the maximal grain size that can be achieved (Ji et al., 2019). Addressing food security could therefore focus on increasing and/or maintaining grain size under adverse environmental conditions as a strategy to improve cereal yields.

## Basic Characteristics Influencing Grain Size

In general, grain size is co-ordinately controlled by cell expansion and proliferation in the developing endosperm and floral tissues (lemma, palea) surrounding the developing grain, determining the ‘sink’ capacity of the grain (Li et al., 2018). In cereals, cell proliferation precedes cell expansion to some extent, beginning at fertilization and ceasing 15–25 days later (Evers and Millar, 2002; Farooq et al., 2014; Li et al., 2018; Bian, 2019). This period of grain development predetermines the maximum size of the grain along the longitudinal and transverse axes and is quite sensitive to abiotic stress; for example in wheat, drought decreases endosperm cell proliferation reducing this ‘sink’ capacity (Setter and Flannigan, 2001). Alternatively, cell expansion relates to the accumulation of dry matter (protein, carbohydrates, and lipids) in the developing grain which is related to photo-assimilate production and transport. This accumulation of dry matter is the major contributor to final grain size and weight and is reported to begin 5–7 days after fertilization and ceases at physiological maturity (Coventry et al., 2003; Sreenivasulu et al., 2004). Adverse conditions during cell expansion primarily damages and/or reduces the photosynthetic area reducing the production and translocation of photo-assimilates to developing grains. Optimization of source–sink pathways therefore represents a promising avenue towards contributing to grain size and weight improvement under both optimal and adverse environmental conditions.

## Genetic Control of Grain Size

In rice, maize, wheat, and barley thousands of quantitative trait loci (QTL) influencing grain size have collectively been detected, yet only a small fraction of underlying candidate genes have been functionally annotated using advanced molecular approaches such as gene cloning with the majority of studies in rice (Ayoub et al., 2002; Huang et al., 2013; Walker et al., 2013; Chen et al., 2016; Yu et al., 2017; Azizi et al., 2019; Li et al., 2019; Wang Q. et al., 2019; Watt et al., 2019). Broadly, the regulatory pathways involved in grain size regulation are represented by: hormone signaling, IKU pathway, G-protein signaling, ubiquitin–proteasome pathway, the mitogen-activated protein kinase pathway and transcription factors (Li and Li, 2016; Azizi et al., 2019). Genes underlying these pathways can be negative or positive regulators of grain size as a result of allele specific epistatic interactions which can substantially influence their effect; for example *OsGLA1* confers a positive effect on grain length and weight driven by a single SNP (Wang T. et al., 2019). Alternatively, *TaDA1* in wheat, a ubiquitin receptor negatively regulates grain size and weight by restricting cell proliferation in the maternal integuments *via* the ubiquitin-proteasome pathway (Liu et al., 2020).

Due to culinary preferences for different sized rice, the majority of grain size research has targeted this staple food crop, particularly the genetic engineering and functional analysis style research. Li et al. (2018) and Azizi et al. (2019) synthesize our current understanding of the pathways and genes involved in grain size regulation in rice which, due to the conservation of gene order and function between the major cereal crop species, is likely to reflect a similar genetic control of grain size in maize, wheat, and barley (Li et al., 2010; Richards et al., 2016). Despite our general understanding of the genetic control of grain size, it is the role of stress inducible transcription factors and their induction that offer promising alternative strategies to maintain and improve grain size and yield under adverse conditions primarily through their stimulation of numerous stress responsive genes (Nakashima et al., 2007; Nutan et al., 2019b).

## Role Transcription Factors Play in Abiotic Stress Tolerance

The ability of a plant to perceive stressful conditions and subsequently respond by inducing stress responsive genes is triggered partly by transcription factors and their interaction with *cis*-acting promoter elements of genes in complex regulatory networks (Gujjar et al., 2014; Rahman et al., 2019). Transcriptional analyses have identified thousands of differentially expressed genes resulting from single and combined abiotic stresses indicating the complexity of stress response and gene expression regulation (Li et al., 2017; Xiong et al., 2017; Osthoff et al., 2019; Pradhan et al., 2019). Multiple transcription factor families have been implicated in abiotic stress response namely the: DREB (dehydration-responsive element binding), ABRE/ABF (ABA-responsive element), MYB (myeloblastosis), NAC, bZIP (basic leucine zipper), and WRKY gene families (Ambawat et al., 2013; Nuruzzaman et al., 2013; Gujjar et al., 2014; Rahman et al., 2019). Often a single transcription factor is able to induce gene expression in response to multiple abiotic stress conditions. The wheat *TaNAC2-5A* transcription factor, for example, is induced by drought, salt, cold, and abscisic acid (ABA) treatment. Overexpression of *TaNAC2-5A* in *Arabidopsis* simultaneously improved drought, salinity, and freezing tolerance (Mao et al., 2012). Interestingly, stress induced *TaNAC2-5A* activity enhanced the expression of *DREB2A* and *ABI5* transcription factors. It has been shown that stress-induced and constitutive overexpression of *DREB2A* in wheat and barley improved tolerance to drought and cold stress due to increased expression of late embryogenesis abundant (LEA) genes encoding dehydrins and cold-responsive proteins that contribute to membrane stability as well as other DREB family genes, further indicating the complexity of stress response and regulatory control (Morran et al., 2011). Similarly, *ZmSNAC1* was found to improve stress tolerance through reduced dehydration, possibly through the NAC-DREB-LEA regulatory module as evidenced in wheat, barley, *Arabidopsis*, and rice (Lu et al., 2012; Hong et al., 2016).

## Role Transcription Factors Play in Grain Size Variation

Aside from the well understood role transcription factors play in plant recognition and response to abiotic stress, numerous studies across the major cereal crop species, particularly rice, have dissected their involvement in grain size modulation (**Table 1**). They co-ordinate cell proliferation and expansion processes not only in the developing grain itself but also in the surrounding floral tissues, lemma, and palea which additionally limit grain size. The antagonistic behavior of two bHLH-type transcription factors (*PGL1* and *APG*) for example, regulates cell elongation in the lemma/palea of rice by the heterodimerization of the two encoded proteins, regulating grain length (Heang and Sassa, 2012). A major gene designated *WIDE AND THICK GRAIN 1* (*WTG1*) in rice encodes an otubain-like protease involved in the ubiquitin–proteasome pathway regulating grain size *via* cell expansion that is reportedly regulated by the transcription factors *ABF1* and *ABI5* (Huang et al., 2017; Li et al., 2018; Zhang et al., 2018). An ortholog in wheat (*TaWTG1-7B*) is highly correlated with the expression of *ABF2* of the ABRE transcription factor family (Zhang et al., 2018).

**Table 1:** List of some key transcription factors involved directly in grain size regulation.

	Locus/gene identity	Transcript factor family & function	Reference
**Barley**	*HvNAC005*	**NAC**. Nutrient remobilization and senescence regulation	Christiansen et al., 2016
	*HvOsbHLH107*	**bHLH**. Regulates cell proliferation in the longitudinal direction, homologous to *OsbHLH107*	Yang et al., 2018
	*HORVU2Hr1G089310*	**MYB**. Orthologous to *OsGL4* which regulates cell elongation in lemma and palea	Wu et al., 2017; Watt et al., 2020
	*Vrs1*	**HD-Zip**. Cell proliferation in the developing lemma/palea contributes to grain length and width variation	Sakuma et al., 2017
**Maize**	*ZmBZR1*	**BZR**. Regulated cell expansion (transverse & longitudinal) *via* cell size genes	Zhang et al., 2020
**Rice**	*OsNF-YC10*	**NF-Y**. Regulates cell proliferation *via* cell-cycle genes and possibly *OsGL7* and *OsGW8*	Jia et al., 2019
	*OsSPL16* (*OsGW8*)	**SBP**. Regulates cell proliferation in the longitudinal and transverse direction by interaction with *OsGW7*	Wang et al., 2015; Liu Q. et al. (2016)
	*OsSPL13 (GLW7)*	**SBP**. Regulates cell elongation in lemma, interacts with *OsSRS5* modifying microtubule formation, grain length variation	Liu Q. et al. (2016)
	*OsGRAS19*	**GRAS**. Regulates brassinosteroid pathway and other regulatory genes (*i.e.* *OsGW8*, *OsGW7*, *OsGL2*)	Chen et al., 2013; Lin et al., 2019
	*OsNAC024*	**NAC**. Positive regulator of GW2, GW5, and D11. Interacts with *OsMED15A* to initiate transcription of above genes	Dwivedi et al., 2019
	*OsGRF4*	**GRF**. Regulates brassinosteroid pathway promoting cell expansion, small influence on proliferation	Che et al., 2015; Hu et al., 2015
	*Os170 (PGL2)*	**bHLH**. Regulates longitudinal cell expansion in lemma/palea by forming heterodimer with APG suppressing activity	Heang and Sassa, 2012
	*OsNF-YC10*	**NF-Y**. Regulates grain width *via* cell division and expansion in the lemma/palea through regulation of cell cycle genes primarily	Jia et al., 2019
**Wheat**	*TaNAM-B1*	**NAC**. Nutrient remobilization and senescence regulation	Uauy et al., 2006
	*TaGLW7*	**SBP**. Orthologous to rice *OsSPL13* regulating grain length	Yang et al., 2019
	*TaABF2*	**ABRE/ABF**. Regulates *TaWTG1-7B* to antagonistically manipulate cell proliferation and expansion. Orthologous to *OsWTG1*	Huang et al., 2017; Zhang et al., 2018

**Figure 1:**
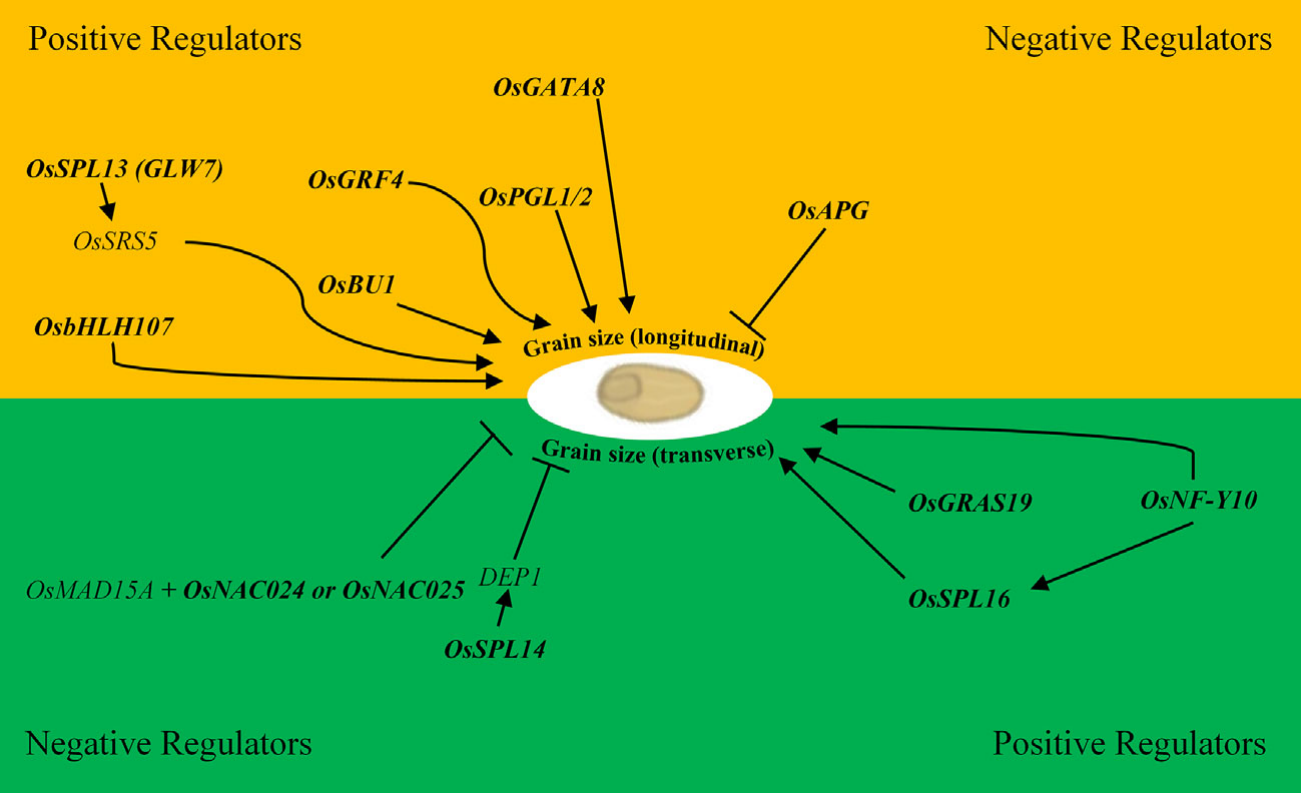
Positive and negative regulatory transcription factors of rice involved in grain size variation in the longitudinal (length; yellow) and transverse directions (width and thickness; green). Bold text represents transcription factors. Arrows represent a positive interaction, and blunt ends are negative interactions i.e. *OsSPL16* positively regulates *DEP1* which in-turn negatively regulates grain width.

The NAC transcription factor family is one of the largest, and numerous NAC genes have been implicated in the control of grain size *via* multiple pathways driven by the diversity of subdomains and their variable protein–protein interactions and DNA-binding activities (Olsen et al., 2005; Dwivedi et al., 2019). The *OsMED15A*-*OsNAC024*/*025* regulatory module for example, positively regulates GW2, GW5 (negative regulators of grain width) and D11 (positive regulator of grain length) in rice through the interaction of the mediator tail subunit of *OsMAD15A* with the two NAC transcription factors that promote the recruitment of additional transcriptional machinery to the promoters of *OsNAC024/025* targets (**Figure 1**) (Dwivedi et al., 2019). In barley, *HvNAC005* transcriptional activity is promoted by the presence of a conserved C-terminal motif reportedly involved in protein–protein interaction suggesting the importance of these types of interactions for the assembly of basic transcriptional apparatus (Christiansen et al., 2016). A signal mediating protein phosphatase 2C (PP2C) is reported to interact with *AtNAP*, a homolog of *HvNAC005*; thus *PP2C* represents a possible mediator initiating transcription of *HvNAC005* targets. Alternatively, the *PP2C*–*SnRK2* ABA-responsive complex is reported to enhance bZIP transcription factor activity in *Arabidopsis* suggesting that this same signaling pathway is active in barley (Hirayama and Umezawa, 2010; Zhang and Gan, 2012).

## Application of Transcription Factors to Manipulate Grain Size Under Stressful Conditions

Despite the diversity of transcription factors, their function, and their involvement in abiotic stress response and regulation of grain size, research marrying the two is surprisingly limited considering the contribution this knowledge could provide to improving cereal productivity. Overexpression of NACs for example, have significantly improved tolerance to drought, salinity, and cold stress in rice and maize (Nuruzzaman et al., 2013). *OsNAC022* overexpression in rice was shown to significantly improve drought and salinity tolerance, although there was a significant negative effect on 1,000-grain weight, a reflection of grain size indicating the importance of identifying suitable transcription factors that can simultaneously improve both stress tolerance and grain size if transcription factors were going to be manipulated for trait improvement (Hong et al., 2016). This study however, drove overexpression *via* the constitutive maize ubiquitin promoter, an approach known to often confer undesirable phenotypes under optimal environmental conditions as was the case for constitutive overexpression of *Ubi1*:*OsNAC6*. It is possible that maintenance of 1,000-grain weight in this instance could have been achieved through the use of a stress inducible and/or tissue specific promoter coupled to *OcNAC022* such as rice *Wsi18* gene promoter which exhibits strong stress induced expression and elevated activity in developing grains specifically (Nakashima et al., 2007; Yi et al., 2011). A GATA-transcription factor, *OsGATA8* has successfully been manipulated, and constitutive expression has been proven to improve drought and salinity tolerance while simultaneously increasing grain length and TGW with no undesirable phenotypes in rice and *Arabidopsis* transgenic lines (Nutan et al., 2019b). It was shown that *OsGATA8* regulated genes involved in reactive-oxygen scavenging enzymes, chlorophyll-biosynthesis enzymes as well as other transcription factors such as *OsDREB1A*.

A WRKY transcription factor *OsWRKY78* positively regulated grain width and was upregulated by ABA and salinity, but downregulated by cold (Zhang et al., 2011). *OsSPL14* positively regulates *DEP1* (**Figure 1**) in-turn influencing grain size, plant architecture, and yield (Jiao et al., 2010; Zhou and Luo, 2013; Stief et al., 2014; Zheng and Qu, 2015; Yue et al., 2017; Watt et al., 2019). Promisingly, *OsSPL14* was recently shown to regulate *OsTB1* which acted as a negative regulator of *OsWRKY94* and suppressed the cold stress induced expression of *OsMADS57* indicating the potential to manipulate *OsSPL14* to simultaneously improve abiotic stress tolerance and grain size (Chen et al., 2018; Nutan et al., 2019a). The R2R3-subclass of the MYB transcription factor family is primarily involved in developmental processes and abiotic stress response compared to the other three subclasses suggesting there is potential to improve both characteristics by focusing on the manipulation of this subclass specifically (Ambawat et al., 2013; Hou et al., 2018). *OsGAMYB* for example, an R2R3-MYB transcription factor involved in gibberellic acid signalling, has been linked to both grain size variation and response to salinity stress possibly through miR159 induction in separate studies (Zhao Y. et al., 2017; Liu et al., 2019). This suggests that it may be possible to manipulate stress response and grain size co-ordinately by targeting transcription factors and/or components of the regulatory network such as microRNAs, the main targets of which are transcription factors (Zhou and Luo, 2013).

## Conclusions and Future Perspectives

The relationship between grain size, yield and the negative effect abiotic stress has on these traits requires further research in order to address the expected threats associated with climate change. The complex regulatory pathways involved in grain size and abiotic stress response suggest that modification of a single transcription factor may offer potential strategies to improving grain size, yield, and abiotic stress tolerance simultaneously as observed by Nutan et al. (2019b). Research needs to address the combined effect of stress response and grain size to transcription factor manipulation to identify on a species-specific level, suitable candidates for trait improvement, something that can currently only be conferred based on a limited number of studies. As between *Arabidopsis* and tomato (Zhou and Luo, 2013), microRNAs which in-turn regulate transcription factors can confer either negative or positive effects on trait expression depending on the species background; in cereals this would necessitate further research, but the relative conservation of gene order and function between the major cereal crop species may enable rapid transferability of knowledge from one cereal species to another. However, care must be taken if a transcription factor is to be manipulated through microRNA modification due to the large gene networks these microRNAs target; miR156 for example, regulates a reported 11 *OsSPL* genes including *OsSPL14* thus the manipulation of miR156 may confer a positive or negative phenotypic response depending on the SPL gene target and necessitates a greater level of understanding (Xie et al., 2006; Wang and Wang, 2015). In addition to alternative targets of transcription factor networks *i.e.* microRNAs, constitutive overexpression of transcription factors as a tool for trait improvement has been proven to induce negative pleiotropic effects on phenotypes in many instances, thus research should address trait response using stress induced and/or tissue specific promoters to improve commercial viability of certain transcription factor modifications.

## Author Contributions

CW came up with the initial concept and wrote the initial draft in conjunction with GZ and CL. All authors contributed to the article and approved the submitted version.

## Funding

CW receives a postgraduate scholarship from the Grains Research and Development Corporation (GRDC), project code UMU1903-003RSX.

## Conflict of Interest

The authors declare that the research was conducted in the absence of any commercial or financial relationships that could be construed as a potential conflict of interest.
